# A Cross-Sectional Comparison of Druggable Mutations in Primary Tumors, Metastatic Tissue, Circulating Tumor Cells, and Cell-Free Circulating DNA in Patients with Metastatic Breast Cancer: The MIRROR Study Protocol

**DOI:** 10.2196/resprot.6024

**Published:** 2016-08-16

**Authors:** Milagros Gonzalez-Rivera, Antoni C Picornell, Enrique L Alvarez, Miguel Martin

**Affiliations:** ^1^Gregorio Marañón Health Research InstituteLaboratory of Translational Oncology, Sequencing & Genotyping-Research Shared ResourcesGregorio Marañón General University HospitalMadridSpain; ^2^Gregorio Marañón Health Research InstituteDepartment of Bioinformatics & BiostatisticsGregorio Marañón General University HospitalMadridSpain; ^3^Gregorio Marañón Health Research InstituteMedical Oncology DepartmentGregorio Marañón General University HospitalMadridSpain

**Keywords:** breast neoplasm, drug therapy, genetics, molecular targeted therapy, sequence analysis.

## Abstract

**Background:**

Characterization of the driver mutations in an individual metastatic breast cancer (MBC) patient is critical to selecting effective targeted therapies. Currently, it is believed that the limited efficacy of many targeted drugs may be due to the expansion of drug resistant clones with different genotypes that were already present in the primary tumor. Identifying the genomic alterations of these clones, and introducing combined or sequential targeted drug regimens, could lead to a significant increase in the efficacy of currently available targeted therapies.

**Objective:**

The primary objective of this study is to assess the concordance/discordance of mutations between the primary tumor and metastatic tissue in MBC patients. Secondary objectives include comparing the genomic profiles of circulating tumor cells (CTCs) and circulating free DNA (cfDNA) from peripheral blood with those of the primary tumor and metastatic tissue for each patient, evaluating these mutations in the signaling pathways that are relevant to the disease, and testing the feasibility of introducing liquid biopsy as a translational laboratory tool in clinical practice.

**Methods:**

The multicenter, transversal, observational MIRROR study is currently ongoing in three participating hospitals. All consecutive patients with MBC confirmed by radiologic findings will be screened for eligibility, either at first relapse or if tumor regrowth occurs while on treatment for metastatic disease.

**Results:**

Patient recruitment is currently ongoing. To date, 41 patients have a complete set of tissue samples available (plasma, CTCs, and formalin-fixed, paraffin-embedded primary tumor and metastatic tumor). However, none of these samples have undergone nucleic acids extraction or targeted deep sequencing.

**Conclusions:**

The results of this study may have a significant influence on the practical management of patients with MBC, and may provide clues to clinicians that lead towards a better stratification of patients, resulting in more selective and less toxic treatments. Additionally, if genomic mutations found in metastatic tissues are similar to those detected in CTCs and/or cfDNA, liquid biopsies could prove to be a more convenient, non-invasive, and easily accessible source of genomic material for the analysis of mutations and other genomic aberrations in MBC.

**Trial Registration:**

ClinicalTrials.gov NCT02626039; https://clinicaltrials.gov/ct2/show/NCT02626039 (Archived by WebCite at http://www.webcitation.org/6jlneVyoz)

## Introduction

Characterization of the driver mutations in an individual metastatic breast cancer (MBC) patient is important for several reasons. First, effective targeted therapies for patients with certain genomic alterations, such as in the human epidermal growth factor receptor 2 (HER2), are available if such a mutation is identified. Second, drugs targeted against molecules such as the estrogen receptor (ER) and specific signaling pathway proteins, such as poly-adenosine diphosphate ribose polymerase, cyclin-dependent kinase (CDK) 4/6, phosphoinositide-3-kinase (PI3K), mammalian target of rapamycin (mTOR), and others are similarly available or under development. Companion diagnostic studies are underway, and are aimed at identifying the specific genomic sequences that can predict the response to these targeted agents, which could aid in the selection of a population of patients who would receive the maximum benefit from specific therapies [1-3]. Third, the expansion of drug-resistant clones with different genotypes compared to the primary tumor, that were already present at the initiation of therapy, may be responsible for the limited efficacy of some targeted drugs in terms of *time to treatment failure*. Identifying the genomic alterations of these clones could indicate that combined or sequential targeted regimens could be used against them, which would lead to a significant increase in the efficacy of currently available targeted therapies.

### Tumor Heterogeneity: Primary Tumor and Metastases

Most cancers are ecosystems of evolving clones with different genotypes [4]; a phenomenon defined in 1976 by Peter Nowell as *tumor heterogeneity* [5]. Tumors arise and develop through a Darwinian clonal evolution [6], and phenotypic heterogeneity is a well-recognized phenomenon described in many cancer types. Numerous comparative genomic hybridization studies, and more recent massively parallel sequencing studies, have generated clear evidence of intratumor heterogeneity in renal and breast cancers [7,8]. Phenotypic heterogeneity was first attributed exclusively to genomic diversity secondary to mutations resulting from clonal evolution. More recently, the study of gene mutations in advanced cancer disease, with acquired resistance to treatments, has evidenced a potential role of the therapy as a selective pressure in the natural clonal evolution of cancer [9-11]. Conversely, the cancer stem cell (CSC) hypothesis postulates that differences between cells can also be due to differences in their differentiation status, which has added an additional perspective to tumor heterogeneity [6].

The initiation and development of cancer are dependent on genetic factors, particularly on the acquisition of multiple *driver* mutations associated with a large number of *passenger* mutations that do not confer any selective advantage. This could, however, be a misleading classification, since passenger mutations might interact with driver mutations in advanced cancer stages and become a reason for resistance to therapy. Additionally, phenotypic heterogeneity may be due to non-genetic factors. According to the CSC hypothesis, phenotypic heterogeneity in cancer is a reflection of differentiation hierarchies that already exist in normal tissues [6]. High expression of stem cell markers, indicating an early stage of differentiation, confers clinically important properties on the tumor cell, such as resistance to therapy and seeding ability.

### Genomic Alterations in Primary Tumors Versus Metastases

An increasing number of studies have identified the existence of genetically distinct clonal subpopulations in some primary tumors [12]. Breast cancers have been classified by means of microarray-based comparative genome hybridization in single cells as either monogenomic or polygenomic, the latter having several clones that are evident in the primary tumor; polygenomic diagnoses carry a poorer prognosis [13,14]. Individual breast cancer tumor cell clones may harbor unique genetic alterations in addition to the founder mutations [15,16]. Distinct populations of breast cancer cells appear to interact in a competitive manner, both in the primary tumor and the micrometastatic environment. The selective pressure of therapeutic interventions can allow resistant cell clones to persist (usually as *dormant* cells) in distant organs. For unknown reasons these clones may expand, first in a silent way and later generating overt metastases, usually several years after the primary diagnosis. A substantial number of subdominant somatic mutations present in the primary tumor can evolve to become dominant in metastatic sites due to a selection process that is a consequence of the therapy [8,10]. Thus, the biology of metastases may be quite different from that of the primary tumor. This phenomenon can be particularly relevant in tumors that relapse after adjuvant treatments, especially if the relapse occurs years after the removal of the primary tumor.

The traditional view of cancer spread and metastasis is that metastatic cells arise from the most aggressive and dominant clone in the primary tumor and, therefore, primary tumors can serve as adequate diagnostic proxies when selecting therapies for metastatic disease. This theory holds true in tumors that are induced by chemicals and those that are associated with short patient survival, such as non-small cell lung cancer [17]; whether this is true in tumors of different etiology, biology, and behavior (such as breast cancer) remains to be established. This traditional view has been challenged by recent studies, in which substantial genetic divergence has been described not only between the primary tumor and metastases, but also between different metastatic sites. The *parallel progression model* considers that breast cancer metastases arise from the primary tumor very early in the evolution of the disease, both evolving in parallel and acquiring different genomic alterations [18]. In reality, it is plausible that both models coexist, with a close clonal relationship between primary tumor and metastases in some breast cancer patients, and marked divergences in others.

Based on these findings, we speculate that breast cancer metastases and primary tumors could harbor both common and unique genomic aberrations. To test this hypothesis, we propose to compare the genomic status of paired breast cancer primary tumors and metastases from the same patients. Obtaining metastatic tumor tissue can be challenging in routine practice, and since different metastatic sites could harbor different alterations, other potential sources of genomic information, such as circulating tumor cells (CTCs) and circulating free DNA (cfDNA), will also be analyzed and compared with the genomic status of the primary and metastatic tumor tissues [19,20]. We will specifically look at the status of the ER/progesterone receptor (PR) pathway, the HER2 pathway, CDK 4/6 activation, phosphatase and tensin homolog, the p53 gene, fibroblast growth factor receptor, and the PI3K pathway, since one or more of these genes/pathways are typically altered in almost all tumors [21].

## Methods

### Study Design and Objectives

This is a multicenter, transversal, observational study performed by the Medical Oncology departments of three hospitals in Madrid, Spain: University Hospital Gregorio Marañón (UHGM), University Hospital Clínico San Carlos, and University Hospital Infanta Cristina. The primary objective of this study is to assess the concordance of mutations and other genomic findings between the primary tumor tissue and metastatic tissue in patients with MBC. Secondary objectives include determining the genomic profiles of CTCs and cfDNA from peripheral blood (liquid biopsy) and comparing these genomic profiles with those from the primary tumor and metastatic tissue for each patient, in order to assess the mutations in the genes of the signaling pathways that are relevant to the disease (ER/PR, HER2, PI3K/RAC-alpha serine/threonine-protein kinase [AKT1]/mTOR, and MAPK pathways, and CDK 4/6 activation) in the various tissues. Additionally, a final objective is to test the feasibility of introducing liquid biopsies as a translational laboratory tool in clinical practice.

### Ethical Considerations and Regulatory Approvals

Approvals for the MIRROR study have been obtained from the ethics committees in all participating hospitals (Study No. Identifier GOMHGUGM092013; ClinicalTrials.gov Identifier: NCT02626039). This study will be carried out in accordance with the guidelines of the Declaration of Helsinki, the principles of Good Clinical Practice as defined by the International Conference on Harmonization (ICH-E6, 17/07/1996), as well as specific regulations in Spain regarding research issues (Spanish Law 14/2007 on Biomedical Research). Before any study procedure, patients will need to provide written informed consent, and allow for the use of tissue samples (metastatic and primary tumor tissues) as well as blood samples for genomic studies and biobanking. Specifically designed case report forms will be used to collect patient information, which will be safely and confidentially stored at the Translational Oncologic Research Unit of each participating hospital.

All recruited patients will follow the therapy prescribed by their oncologists, according to individual clinical practices. No additional treatment or change in treatment is required for participation in this study.

### Patient Selection

All consecutive patients with MBC who visit the Oncology Services of the participating hospitals will be screened for eligibility. To participate in the study, patients must be over the age of 18 with MBC (all subtypes) confirmed by radiologic findings, either at first relapse or if tumor regrowth occurs while on treatment for metastatic disease since November, 2013. Additionally, patients will have a formalin-fixed paraffin-embedded (FFPE) tissue sample isolated from the primary tumor, and a clinical indication for biopsy of the metastatic relapse. Patients will be excluded if they are unable or unwilling to give informed consent, have metastatic bone disease only, have coagulation disorders, are unable to have peripheral blood drawn, or have an Eastern Cooperative Oncology Group performance status of 3 or 4.

Patients who sign the informed consent form and enter into the study, but fail to undergo the biopsy (or for whom primary tumor tissue is not available), will be ineligible and considered recruitment failures. Patients for whom biopsies are not successful due to failure of tumor tissue retrieval will be also considered recruitment failures.

### Study Procedures

After the patient is enrolled, baseline demographic characteristics, medical history, and a complete physical examination will be recorded, and metastatic tissue and blood specimens will be obtained. Additionally, a paraffin block of the primary tumor tissue will be obtained and archived. Investigators will be trained in the specimen collection protocol of the Translational Oncology Research Unit of UHGM for the genomic studies. Biopsies of both the metastatic tissue and the blood sample must be taken close together in time; if the metastatic tumor tissue is obtained by puncture, the blood sample must be taken no more than 15 days before or after the biopsy. If the biopsy procedure is surgical, a peripheral blood sample must be taken on the same day as (or the day before) the biopsy procedure. Biopsy procedures will be guided using imaging technology as per clinical practice, unless metastasis is cutaneous. The biopsied tissue will then be formalin-fixed and sent for examination by the Pathology Service of the participating hospital. Whenever possible, a separate sample of the tissue will be sent directly to the Translational Oncology Research Unit of UHGM within 24 hours.

At the time of the biopsy, an assessment of any adverse events (AEs) possibly related to the biopsy procedure will be recorded. Those patients who do not return for a biopsy follow-up visit will be contacted by telephone for an AE safety evaluation during the 20 days following their most recent biopsy procedure. All AEs suspected to be related to the biopsy procedure will be followed up weekly, or as clinically indicated, until resolution or stabilization.

Patients (or parents/guardians in the case of disabled participants) may voluntarily withdraw from the study or be lost to follow-up, and patients may be dropped from the study at the discretion of the investigator at any time.

### Biobanking and Sample Analysis

All specimen analyses will be performed at the laboratory of the Translational Oncology Research Unit of UHGM. For each patient, five types of biological samples will be obtained and analyzed, namely FFPE from primary tumor, FFPE from one metastatic site, plasma, white blood cells (WBCs), and CTCs. Parameters to be studied in each specimen are shown in Table 1.

**Table 1:** Genomic studies per sample and specimen.

Specimen	Sample	Study	Biobanking
Formalin-fixed paraffin-embedded primary tumor	Tissue section	Immunohistochemistry for ER/PR, HER2, P53, Ki67	Formalin-fixed paraffin-embedded block
	DNA	Mutation profile and copy number variation	DNA
	RNA	PAM50 intrinsic subtype	Aliquot for RNA sequencing
Formalin-fixed paraffin-embedded metastases	Tissue section	Immunohistochemistry for ER/PR, HER2, P53, Ki67	Formalin-fixed paraffin-embedded block
	DNA	Mutation profile and copy number variation	DNA
	RNA	PAM50 intrinsic subtype	Aliquot for RNA sequencing
White blood cells	DNA	Mutations and copy number variation in germinal line	DNA
Circulating tumor cells	Circulating tumor cells	Number	DNA
	RNA	PAM50 intrinsic subtype	Aliquot for RNA sequencing
	DNA	Mutation profile and copy number variation	DNA
Plasma	Circulating free DNA	Quantification, mutation profile, and copy number variation	DNA

For primary tumor and metastatic tissues, a hematoxylin and eosin section of FFPE sample will be assessed by the pathologist, with the aim of selecting areas of tumor with >70% cellularity. Two unstained sections will be macrodissected and processed for both DNA extraction using the QIAamp DNA FFPE Tissue Kit (Qiagen) and RNA extraction using the RNeasy FFPE kit (Qiagen). Somatic mutations will be assessed by targeted deep sequencing applied to plasma DNA (cfDNA test), tumor DNA (sDNA test) and, CTC-DNA (ctcDNA test). For each patient, genomic DNA from normal peripheral blood leukocytes will be assayed. Fully customized amplicon-based assays will be used to include genes of the ER/PR, HER2, PI3K/AKT1/mTOR, and MAPK pathways, and CDK 4/6 activation. The somatic mutation profile of the DNA extracted from all samples will be assessed according to the techniques developed as proof of concept by Forshew [20], Dawson [19], and Mutarza [22]. Structural variants of DNA will be assessed by applying the copy number variation (CNV)/CNV-FFPE nCounter Analysis System. RNA will be assayed for the PAM-50 expression profile to determine the subtype of breast cancer in the primary tumor, while the metastatic tissue and CTCs will be analyzed using the nCounter Analysis System.

Blood collected in ethylenediaminetetraacetic acid tubes will be processed within one hour to separate plasma, WBCs, and CTCs. Samples will be frozen at -80ºC until the nucleic acids extraction is scheduled. Following the method described by Dawson et al [19], DNA will be extracted from plasma using the QIAamp Circulating Nucleic Acid kit (Qiagen). The total amount of cfDNA will be quantified using previously described methods [19,23]. After collection of plasma, the remaining buffy coat-containing WBCs will be removed. DNA will be extracted using adsorption methods. Peripheral blood mononuclear cells will be obtained from blood by density gradient centrifugation, which will then be mixed with CTC beads and loaded onto a microfluidic cartridge following the ISOFLUX-CTC procedures (Fluxion Biosciences Inc). Enriched CTCs will be processed for enumeration or be stored for further DNA and RNA extraction.

### Sample Size and Statistical Analyses

Analyses of genomic DNA sequencing data are considered exploratory in nature, and will generate new hypotheses. For this study, we have estimated that three patients per month will be enrolled, reaching a final sample size of 40 patients with complete data to proceed for further analyses.

According to Hart et al [24], a sample size calculation comparing two groups involves five factors, namely (1) the depth of sequencing and consequent expected count for a given transcript, (2) the coefficient of variation of counts within each of the two groups, (3) the fold-change that we wish to detect, (4) the target false positive rate and the false negative rate, and (5) the sample size in each group. Setting 3000x as the sequencing depth and 0.4 as the coefficient of variation, we estimate there is enough statistical power (80%) to detect effects higher than 1.29.

Clinical and demographic characteristics of patients will be described using descriptive statistical methods. Univariate analysis will be performed in order to describe the distribution, central tendency, and dispersion of the variables. Bivariate analysis will be used to find relationships between different variables. Analyses will be performed using the Statistical Package STATA version 12.1 or the newest version of R, according to the requirements of the analyses. Additionally, a scoring algorithm will be established in order to compare the mutation/CNV status in cfDNA/CTC-DNA against primary tumors, metastases, and germinal line DNA.

The genome sequencing data will be analyzed using bioinformatics tools. Sequencing reads will be aligned to the human reference genome building hg38 (GRCh38) to generate a list of potential genomic variations through variant-calling procedures. The alignments will be run using tools based on the Burrows-Wheeler Transform such as Burrows-Wheeler Aligner or Bowtie2. The aligned results will be processed using the SAMtools suite in order to obtain sorted, indexed, and binary files. If necessary, duplicate reads will be marked with Picard and removed before single nucleotide variation (SNV) identification with softwares such as SAMtools, mpileup, and MuTect. These programs will be used to detect low-frequency variants, as data cannot be expected to follow normal ploidy models. Additional steps will be performed if needed, such as the recalibration and realignment using BaseRecalibrator and IndelRealigner, both in the GATK suite. In recent years, a number of important algorithms and implementations have been designed for specific SNV detection tasks in matched samples. Depending on the number and nature of the detected SNVs, it may be appropriate to filter the results in order to keep only those located in exon junctions, or those leading to nonsynonymous changes. Moreover, germinal DNA will be taken as a control to assess background noise, and establish sample-specific thresholds for SNVs to be accepted. Identified and confirmed SNVs will be annotated with broadly-used tools such as SIFT, Variant Effect Predictor, or MutationAssessor. Finally, self-contained pathway and functional analyses will be applied to the obtained results, in order to check that a specific pathway has different genomic or expression patterns between compared samples. The high throughput sequencing raw data will be stored as clinical research files for further reevaluations. All reference genomic files containing the genes, transcripts, and annotated mutations will be used as the control genomic profile of each sample.

## Results

**Figure 1:**
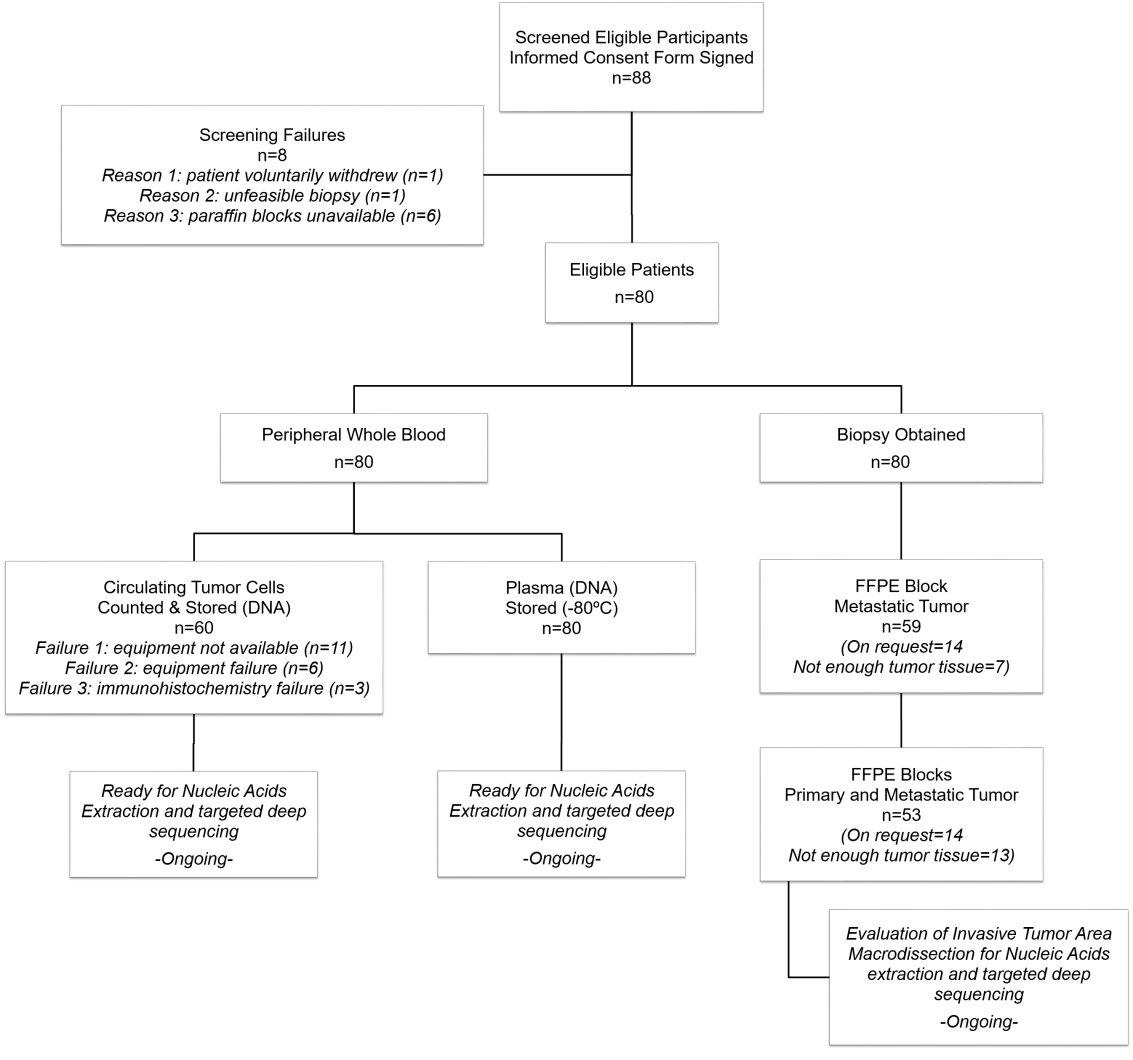
Flowchart of participants, specimens, and samples through the study.

Patient recruitment started in November, 2013 and is currently ongoing. To date, 41 patients have a complete set of tissue samples available (plasma, CTCs, and FFPE of primary tumor and metastatic tumor). However, none of these samples have undergone nucleic acids extraction or targeted deep sequencing. Due to the high standards of the quantity and quality of DNA required to perform these procedures, the recruitment period is still open (it is expected that some of the tissue samples will not be valid). Final study results are expected to be available in December, 2016. Additional information regarding the progress of this study is described in the Figure 1.

## Discussion

The MIRROR study is one of the first observational studies to test the hypothesis that breast cancer metastases and primary tumors harbor different genomic profiles (related to genomic regions of interest) in a clinically relevant proportion of MBC patients, while concurrently assessing whether genomic aberrations found in the metastatic tissue are detectable in CTCs and cfDNA. Our study is a descriptive observational trial in the clinical setting. Genomic data will be described along with the clinical course of the disease for each patient. Therefore, data will include the type and the time of recurrence, and the different therapies received until the biological samples were collected.

Most registration trials examining new targeted agents involve companion diagnostic studies that are implemented in parallel with the efficacy study. In the majority of these studies, tumor biopsies are required prior to enrollment in order to characterize the genomic status of the patient. However, for practical reasons, some studies are accepting primary tumors as appropriate material for genomic testing. When biological samples of the current disease status are not available, two hypotheses have to be assumed: (1) all the genomic aberrations that are present in the metastases were already present in the primary tumor, and (2) all metastatic sites are genomically homogeneous. However, these hypotheses may not be true or, more precisely, they could be true in some patients but not in others. In this study, we intend to shed some light onto this issue.

The results of the present study may have a significant influence on the practical management of patients with MBC, and may guide clinicians towards a better stratification of patients, resulting in more effective and less toxic treatments. Additionally, if genomic mutations found in metastatic tissue are similar to those detected in CTCs and/or cfDNA, liquid biopsies could prove to be a more convenient, non-invasive, and easily accessible source of genomic material for the analysis of mutations and other genomic aberrations in MBC.

## Abbreviations

AE: adverse event
AKT1: RAC-alpha serine/threonine-protein kinase
CDK: cyclin-dependent kinase
cfDNA: circulating free DNA
CNV: copy number variation
CSC: cancer stem cell
CTCs: circulating tumor cells
ER: estrogen receptor
FFPE: formalin-fixed paraffin-embedded
HER2: human epidermal growth factor receptor 2
MBC: metastatic breast cancer
mTOR: mammalian target of rapamycin
PI3K: phosphoinositide-3-kinase
PR: progesterone receptor
SNV: single nucleotide variation
UHGM: University Hospital Gregorio Marañón
WBC: white blood cell
